# Smokeless tobacco and oral cancer in the Middle East and North Africa: A systematic review and meta-analysis

**DOI:** 10.18332/tid/110259

**Published:** 2019-07-18

**Authors:** Mir Faeq Ali Quadri, Santosh Kumar Tadakamadla, Tenny John

**Affiliations:** 1 Faculty of Dentistry, Jazan University, Jazan, Saudi Arabia; 2 School of Dentistry and Oral Health, Griffith University, Southport, Australia; 3 Menzies Health Institute Queensland (MHIQ), Southport, Australia

**Keywords:** systematic review, meta-analysis, smokeless tobacco, oral cancer, shammah

## Abstract

**INTRODUCTION:**

Cancer of the oral cavity is regarded lethal with a fairly low mean 5-year survival rate. The current systematic review and meta-analysis is the first of its kind to examine, if the evidence from the Middle East and North African region indicates an association between oral cancer and tobacco; and evaluates the quality of the evidence that portrays this relationship.

**METHODS:**

A search for articles was carried out in October 2017 and then cross-checked at the end of June 2018 using Medline, Web of Science, CINAHL, and Cochrane databases. Retrieved articles were later subjected to eligibility criteria. The search was not limited to any particular research design adopted by the investigators. However, dissertations, theses and opinion-based reviews generated from the search were excluded during the screening of titles and abstracts. Quality of included studies was determined objectively (Newcastle Ottawa Scale) and subjectively. Revman (Version 5.3) was used for conducting the meta-analysis.

**RESULTS:**

Six studies satisfied the selection criteria of the current review. The New Castle Ottawa evaluation scale suggested that the three cross-sectional studies and the three case-control studies included in the current review were of relatively low to moderate quality. All included studies explored the association of only one form of smokeless tobacco, i.e. shammah. Three case-control studies revealed a pooled estimate odds ratio of 38.74 (95% CI: 19.50–76.96), indicating that the odds for the occurrence of oral cancer among shammah users were nearly 39 times higher compared to the non-users.

**CONCLUSIONS:**

Shammah is a potential risk factor for oral cancer; thus, it is necessary that public health practitioners design and implement effective strategies to prevent the abuse of shammah.

## INTRODUCTION

The global annual incidence rate of 354864 new cases of oral cancer (oral cavity and lip cancer) makes it one of the most common non-communicable diseases^1^. Some epidemiological studies have considered it to be the sixth most prevalent cancer and one of the foremost contributors towards mortality^2,3^. The reputation of being lethal is also demonstrated by its low mean 5-year survival rate, with many of the deaths from the developing nations^2,4,5^. Such nationwide datasets provide good insight on the prevalence of the disease and its associated risk factors, but region-specific studies on oral cancer (OC), as suggested in the first meeting of the Global Oral Cancer Forum, are also equally important^6^. The existing disparity of disease prevalence and associated risk factors among populations residing in different regions of the globe could be explained in a better way through region-specific studies, which add more weight to the findings as they consider and control for common contributing factors like genetics, habits, and lifestyle. Some regions like Europe, Oceania and North America have population-based cancer registries, which cover a large part of their residents, while the same is yet to be completely followed by South-East Asia and Middle Eastern regions^7^. Thus, region-specific studies will enhance the provision of comparative data from representative samples.

One such region with a history of confined culture and heritage is the Middle East and North Africa (MENA). According to the World Bank, there are 19 countries in the MENA region with populations similar in many characteristics^8^. The countries that belong to the MENA region are: Algeria, Bahrain, Djibouti, Egypt, Iran, Iraq, Jordan, Kuwait, Lebanon, Libya, Morocco, Oman, Qatar, Saudi Arabia, Syria, Tunisia, United Arab Emirates, West Bank and Gaza Strip, and Yemen. Reports have predicted an increased oral cancer burden in the MENA region, attributed to population growth, ageing population, diagnostic methods, and most importantly due to excessive substance abuse^9,10^.

The World Health Organization on its website affirms that the heavy use of tobacco is one of the main contributing factors towards the growing incidence of oral cancer^9^. Depending on the method of consumption, tobacco is broadly divided into smoked tobacco and smokeless tobacco. Smokeless tobacco is not burnt but is most often snuffed, chewed, or dipped^11^. However, there are more than twenty-eight different varieties of smokeless tobacco being consumed globally^12^, and in the MENA region it is known through different names such as: shammah, toombak, maras, nass and neffa^13^. Consumers, who are reportedly adult males^14-16^, place the orally used smokeless tobacco either in their buccal mucosa, labial mucosa or under the tongue, and then suck (dipped) or chew it on a timely basis^11^.

Smokeless tobacco is equally addictive as smoked tobacco^17-19^, and its association with oral cancer is demonstrated by systematic reviews from the Asia Pacific region^20^, Europe, and North America^21^. However, there is no systematic review from the MENA region on this relationship. We postulate that the studies conducted in the MENA region provided low quality evidence to support this relationship. Thus, the objectives of this study are: 1) examine if the evidence from the MENA region indicates an association between oral cancer and smokeless tobacco, and 2) evaluate the quality of evidence that portrays this relationship.

## METHODS

### Permission and registration

The framework of this review was based on the guidelines provided by the Preferred Reporting Items for Systematic Reviews and Meta-Analyses (PRISMA)^22^. The protocol was registered with the International Prospective Register of Systematic reviews and was assigned the following identification code: PROSPERO–CRD–42018093137.

### Eligibility criteria

All publications in English language journals demonstrating the relationship between oral cancer and smokeless tobacco in the MENA region were included. The search of published articles was not limited to any particular research design adopted by the investigators. However, dissertations, theses and opinion-based reviews generated from the search were excluded during the screening of the titles and abstracts. Research studies conducted on human participants residing in the MENA region, irrespective of sex, age and socioeconomic status were included in this review. Studies performed on expatriate populations residing in the MENA region were not included as the habit of tobacco use as well as the type of tobacco consumed may vary between the local residents and the expatriates^23^.

### Exposure and outcome

Exposure comprised any form of smokeless tobacco reported to be consumed, irrespective of the method (chewing, dipping etc.) of consumption, duration of the habit, or frequency. The outcome was histopathologically confirmed cancer cases of the oral cavity. Premalignant lesions and any other non-confirmed oral lesions were not considered as only a small percentage of them progress into malignant cases on detection^24,25^. The PICO/PECO question for this review was: ‘Is oral cancer (Outcome) more common in smokeless tobacco users (Exposure) in comparison to non-users (Comparator) among the residents of the MENA region (Participants)?’.

### Information sources and search strategy

Search for articles was carried out in October 2017 and then updated at the end of June 2018 using Medline, Web of Science, CINAHL, and Cochrane databases. Multiple searches were performed using various combinations of keywords including the Medical Subject Heading terms (Table 1). Reference lists of the included studies were also screened for relevant articles.

**Table 1:** Demonstrating the search terms used in retrieving the reports

1 Shamma OR Shammah OR Snus OR Tobacco OR Toombak OR Maras OR Neffa
2 Oral cancer OR Mouth cancer OR Mouth neoplasm OR Oral neoplasm OR Oral squamous cell carcinoma OR Head and neck cancer OR Cancer of mouth OR Head and neck neoplasm
3 Algeria OR Bahrain OR Djibouti OR Dubai OR United Arab Emirates OR Egypt OR Iran OR Iraq OR Jordan OR Kuwait OR Lebanon OR Libya OR Morocco OR Oman OR Palestine OR Qatar OR Saudi Arabia OR Syria OR Tunisia OR West Bank and Gaza OR Yemen OR Middle East OR North Africa

### Study selection and data extraction

Reviewers (SKT and MFAQ) independently performed article searches using search strings given in Table 1. No discrepancies in the list of articles were detected after removing the duplicates. The third reviewer (TJ) screened the titles and abstracts of the retrieved articles to identify the relevance of the reports to the objectives of this review. Subsequently, full texts of these included articles were reviewed to assess their eligibility for final appraisal and synthesis.

Data extraction from the final list of included studies was performed using a predesigned form. The information gathered was as follows: name of author(s), year of publication, study location, study design, sample size, type of smokeless tobacco, method of assessment of the variables and results on the association of smokeless tobacco with oral cancer as stated by the authors.

### Quality assessment of included studies

Subjective and objective methods were used to report on the quality assessment of the included studies. Objective assessment involved the Newcastle Ottawa Scale for observational studies. The scoring was done under three headings: Selection, Comparability, and Outcome. The seven items under these headings were marked accordingly if they satisfied the required criteria for that particular item. The subjective assessment to report on the quality of the published studies involved critical appraisal of articles by one author (MFAQ) and a subsequent cross-check by another author (SKT) using criteria proposed by Hermont et al.^26^.

### Quantitative assessment

Meta-analysis was conducted for the three case-control studies that assessed the association between shammah and oral cancer using Revman (Version 5.3)^27^. Odds ratio was the summary estimate in all the three case-control studies. To make use of adjusted odds ratios as summary estimates from the included studies, generic inverse variance method was used. As the studies varied in their characteristics, a random effects model and inverse variance method was used. I^2^ statistic was calculated to evaluate the heterogeneity between the studies. Interpretation of I^2^ was based on the Cochrane handbook: with I^2^<40% categorized as not important, while 30–60%, 50–90%, and over 75%, were considered as moderate, substantial, and considerable, respectively. Although these categories overlap, the importance of the I^2^ value depends on the magnitude and direction of effects and the strength of heterogeneity^28^. We intended to use Funnel plots and Egger’s regression test to evaluate the publication bias between the studies only if a sufficient number of reports were available.

## RESULTS

### Identification and screening

**Figure 1:**
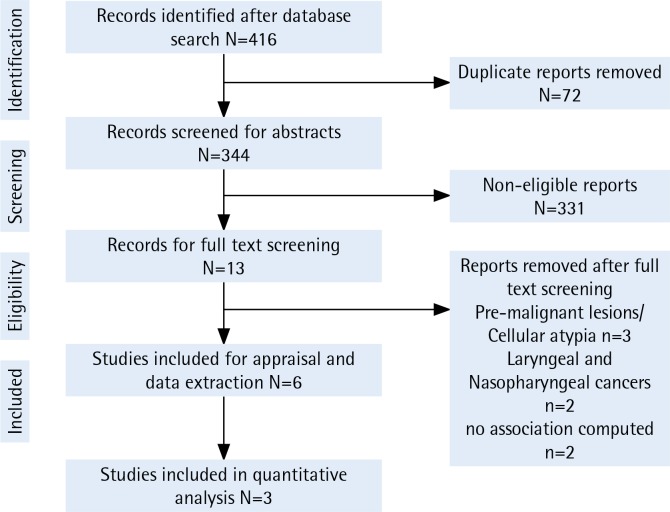
PRISMA flowchart demonstrating the reports identified, screened, and included in the review

Electronic search displayed 416 hits from PubMed via Medline, Web of Science, CINAHL, and COCHRANE databases. Around 72 duplicate titled reports were removed before screening the abstracts. A total of 344 abstracts were subjected to the inclusion and exclusion criteria resulting in 13 articles remaining. The full text of these articles (n=13) were read in order to identify the relevance to the current review. Three articles did not have findings on oral malignancies^29-32^ of which three reported on premalignant lesions and one reported on cellular atypia. Two studies had not statistically computed the association of smokeless tobacco with oral cancer^33,34^. The final two excluded studies had a lower number (n=2) of oral cancer cases^32^ and no identified smokeless tobacco users^35^. Thus, the end synthesis had 6 articles (Figure 1) with three available for meta-analysis.

### General description of the retrieved studies

Most of the included studies (n = 5) had findings from Saudi Arabia, followed by one study from Yemen. All included reports had clear objectives to analyze the relationship between smokeless tobacco and oral cancer. Three studies adopted a cross-sectional design (Table 2) to address the relationship, while the other three studies adopted a case-control design (Table 3). Total cases of oral cancer were 549; with most of the study participants being old (Tables 2 and 3). Findings extracted from the studies are discussed under two headings pertaining to the design of the research. This was done in order to have a clear interpretation of results from each study.

**Table 2:** Main characteristics of the cross-sectional studies

*Author Year Country*	*Sample size*	*Method of OC diagnosis*	*Measure of SLT and Adjusted variables*	*Results and Conclusion*
Salem et al.^37^ 1984 Saudi Arabia	Total Sample Size = 661 Total SCC cases = 7 Carcinoma in situ = 2	Biopsy was performed and dysplastic changes were observed as defined by WHO	Measure = Questionnaire Type = Shammah Duration = NA Frequency = NA Adjusted variable = Smoking	**Results** Almost all the individuals with oral mucosal lesions had been using shammah persistently for more than 5 years, whereas none of those who only smoked cigarettes developed a lesion. The mucosal lesions were found always to be present at the site where shammah was habitually held. **Conclusion** A causal relation exists between the use of shammah and the development of lesions of the oral mucosa.
Amer et al.^38^ 1985 Saudi Arabia	All patients between 1 June 1981 and 30 July 1983, from King Faisal Specialist Hospital and Research Centre Squamous cell carcinoma cases = 68	Histopathology (TNM Staging)	Measure = Questionnaire Type = 49% admitted to the use of shammah Mean duration = 27.1 years Frequency = NA Adjusted variable = NA	**Results** 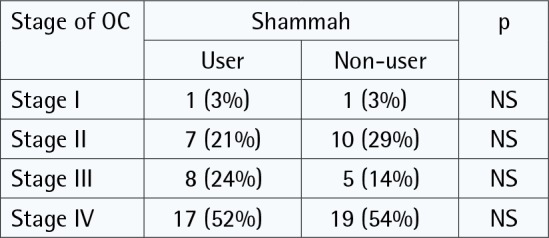 **Conclusion** No conclusion on the relation between OC and shammah was mentioned. **Results** 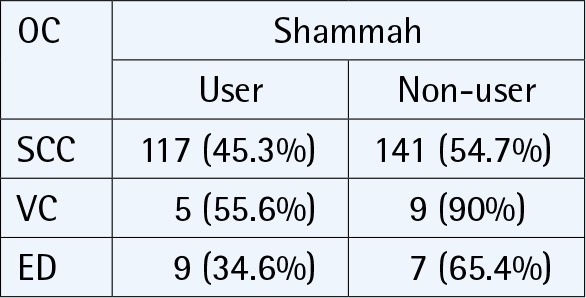
Saleh et al.^39^ 2017 Saudi Arabia	Total patient data for oral lesions = 714 Malignant lesions = 38.8% Squamous cell carcinomas = 258 Verrucous carcinoma = 10 Epithelial dysplasia = 26	Hospital records from surgical biopsy unit	Measure = Hospital records Type = Shammah Users = 45.3% Non-users = 54.7% Duration = NA	**Conclusion** The number of oral and maxillofacial biopsied lesions documented in this study was much lower than expected, which can be explained by the lack of adequate biopsy services for oral health care in this region. Furthermore, the patterns and types of these lesions and their associated oral habits should be documented for planning appropriate dental health services.

OC: oral cancer, SLT: smokeless tobacco, NA: not assessed, SCC: squamous cell carcinoma, VC: verrucous carcinoma, ED: epithelial dysplasia.

**Table 3:** Main characteristics of the case-control studies

***Author Year Country***	Nasher et al.^40^ 2014 Yemen	Quadri et al.^16^ 2015 Saudi Arabia	Al Harbi and Quadri^42^ 2018 Saudi Arabia
***Sample size***	Number of cases = 60 Number of controls = 120	Number of cases = 48 Number of controls = 96	Number of cases = 70 Number of controls = 140
***Definition of cases***	Histopathologically graded as well differentiated, moderately differentiated or poorly differentiated	Hospital records searched for histopathology	Hospital records searched for histopathology
***Definition of controls***	Controls were selected from among patients of same hospital seeking dental treatment for conditions other than mucosal lesions. Matched = Age, gender, location	Controls were patients visiting the hospital following the same referral route as cases approaching various departments other than that of Oral surgery, E.N.T and Oncology. Matched = Age, gender, location	Controls were defined as subjects free of OC and who followed the same referral route as cases, except that they had approached departments other than that of Oral surgery, Otorhinolaryngology–Head and Neck Surgery and Oncology. Matched = Age, gender, location
***Independent variables assessed***	History of current and previous qat (Catha edulis) chewing, smoking, dipping tobacco (shammah) use and alcohol consumption was obtained by direct questioning of the study subjects.	Apart from demographic details of each participant, shammah use – its duration, qat chewing, cigarettes (number of packs per day) and pipe smoking were the variables that were recorded through a questionnaire.	Explanatory variables were further dichotomized as ‘ever users’ and ‘never users’. Ever users were considered as subjects who frequently consumed these potential risk factors; whereas ‘never users’ were subjects that had never consumed these substances in their lifetime.
***Statistical method***	**Statistical method** = Multiple logistic regression	**Statistical methods** = Multivariate analysis; Multiple logistic regression	**Statistical methods** = Multiple logistic regression
***Results and Conclusion***	**Results** Model 1 Pseudo R^2^=0.392 Ex shammah use: (OR=12.6; 95% CI: 3.3–48.2, p<0.001) Current shammah use: (OR=39; 95% CI: 14–105, p<0.001) Ex smoking: (OR=0.11; 95% CI: 0.03–0.37, p<0.001) Reference group: non-users Model 2 Pseudo R^2^=0.358 Shammah use: (OR=149.5; 95% CI: 12.3–1812, p<0.001) Shammah + qat: (OR=43.1; 95% CI: 7.0–266, p<0.001) Shammah + qat + smoking: (OR=14.2; 95% CI: 2.9–69.0, p<0.001) **Conclusion** The study demonstrates that shammah use is a major risk factor for oral cancer in Yemen, while providing little evidence for the involvement of smoking, alcohol drinking, qat chewing or HPV or EBV infections.	**Results** Model 1 Pseudo R^2^=0.4 Shammah: (OR=29.30; 95% CI: 10.33–83.13, p<0.05) Cigarette: (OR=6.74; 95% CI: 2.18–20.83, p<0.05) Model 2 Pseudo R^2^=0.41 Shammah: (OR=37.24; 95% CI: 12.25–113.18, p<0.05) Cigarette: (OR=10.48; 95% CI: 2.88–3.11, p<0.05) Qat + shammah: (OR=0.01; 95% CI: 0.00–0.65, p<0.02) **Conclusion** The study reveals that shammah is the major cause of oral cancer in the region.	**Results** Model 1 R^2^=0.4, p=0.03 Shammah: (OR=33.01; 95% CI: 13.01–76.31) Shisha: (OR=3.96; 95% CI: 2.11–28.83) Model 2 R^2^=0.47, p=0.02 Ever shammah + ever shisha: (OR=35.03; 95% CI: 11.50–65.66) Ever shisha + ever cigarette: (OR=10.52; 95% CI: 1.03–33.90) Ever shammah + ever cigarette: (OR=10.10; 95% CI: 0.50–20.40) **Conclusion** It is seen that the combined use of risk factors like shammah, shisha and cigarettes have serious implications on the onset of OSCC.

OC: oral cancer, SLT: smokeless tobacco, OR: odds ratio.

### Cross-sectional studies

Salem et al.^36^, in 1984, indicated a relationship between smokeless tobacco and seven squamous cell carcinoma cases and two carcinoma-in-situ cases from the Gizan region of Saudi Arabia. More specifically, they had mentioned that all the confirmed squamous cell carcinoma cases were using shammah (a form of smokeless tobacco) for many years. Later, Amer et al.^37^ in 1985 published their findings on 68 histopathologically confirmed squamous cell carcinoma cases of oral cavity in Saudi Arabia. In their study, it was reported that 49% of the confirmed cases admitted regular use of shammah. The authors also stated that the actual number of users could be higher, as the clinical findings showed some evidence of shammah use despite denials by the subjects^37^.

The mean age for an individual to begin the habit of shammah use, according to their study, was 24.3 years and the duration of shammah use was 10–50 years. Nearly 73% of these individuals were from low socioeconomic environments in the Gizan region of Saudi Arabia^37^. The end result from their study, after group comparison, did not show any significant variation (p>0.05) in oral cancer cases between users and non-users of smokeless tobacco. In the study of Saleh et al.^38^, higher percentage of oral cancer cases were attributed to smokeless tobacco use, but it was observed that the numerical data in their tables did not match this interpretation (Table 2). To summarize, these cross-sectional studies demonstrated the relationship between shammah and oral cancer, but the findings were limited to the nature of the study design.

### Case-control studies

The first case-control study of the MENA region was conducted in Yemen by Nasher et al.^39^ with 60 squamous cell carcinoma cases and 120 controls. It revealed that the subjects using shammah demonstrated higher odds (OR=149.5; 95% CI: 12.3–1817.25) of oral cancer than non-users. Later, Quadri et al.^16^ reported that shammah users had nearly 37 times (OR=37.24; 95% CI: 12.25–113.21) higher odds of developing oral cancer in comparison to non-users (Table 3). The very recent study by Al Harbi and Quadri^40^, in 2018, reported that shammah users had 33 times greater odds of oral cancer than nonusers (OR=33.01; 95% CI: 13.01–83.76).

### Quality assessment of included studies

Quality assessment was conducted objectively using the Newcastle Ottawa Scale with the points mentioned earlier and the detailed report is presented in Tables 4 and 5. In terms of sample representativeness among the cross-sectionally designed studies, it was seen that Salem et al.^36^ did not provide a representative sample; in fact their study recruited only a selected group of shammah users. Two other studies had ‘a somewhat representative’ sample as the oral cancer cases were recruited from the tertiary care centers of their respective regions^37,38^. With regard to sample size, it is seen that one published research did not have any report on sample size calculation^36^ while the other two studies had provided justification for their included sample sizes^37,38^. Lastly, it is to be noted that none of the studies had reported on the non-respondents in their respective studies (Table 4).

**Table 4:** Newcastle Ottawa Scores for the cross-sectional studies

*Reference*	*Selection*				*Comparability*	*Outcome*		*Quality of research*
	*Representativeness of the sample*	*Sample size*	*Non-respondents*	*Ascertainment of exposure*		*Assessment of the outcome*	*Statistical test*	
Salem et al.^37^ 1984	c	b	b	a**	b*	b**	b	Low
Amer et al.^38^ 1985	b*	a*	b	b*	b*	b**	a*	Moderate
Saleh et al.^39^ 2017	b*	a*	b	b*	b*	b**	b	Low

Representativeness of the sample: a) Truly representative of the average in the target population, all subjects or random sampling*, b) Somewhat representative of the average in the target population, non-random sampling*, and c) Selected group of users. Sample size: a) Justified and satisfactory*, and b) Not justified. Non-respondents: a) Comparability between respondents and non-respondent characteristics is established, and the response rate is satisfactory*, and b) The response rate is unsatisfactory, or the comparability between respondents and non-respondents is unsatisfactory. Ascertainment of the exposure (risk factor): a) Validated measurement tool**, and b) Non-validated measurement tool, but the tool is available or described*. Comparability (maximum 2 stars): b) The study control for any additional factor*. Outcome (maximum 3 stars): b) Record linkage**. Statistical test: a) The statistical test used to analyze the data is clearly described and appropriate, and the measurement of the association is presented, including confidence intervals and the probability level (p-value)*, and b) The statistical test is not appropriate, not described or incomplete.

**Table 5:** Newcastle Ottawa Scores for the case-control studies

*Reference*	*Case definition*	*Representation*	*Control selection*	*Control definition*	*Comparability*	*Exposure ascertainment*	*Quality of research*
Nasher et al.^40^	a	B	b	A	**	**	Moderate
Quadri et al.^16^	a	B	b	A	**	**	Low
Al Harbi and Quadri^42^	a	A	b	A	**	**	Moderate

Case definition adequate?: a) Requires some independent validation (e.g. >1 person/record/time/process to extract information, or reference to primary record source such as x-rays or medical/hospital records). Representativeness of the cases: a) All eligible cases with outcome of interest over a defined period of time, all cases in a defined catchment area, all cases in a defined hospital or clinic, group of hospitals, health maintenance organization, or an appropriate sample of those cases (e.g. random sample), and b) Not satisfying requirements in part a), or not stated. Selection of controls: a) Community controls (i.e. same community as cases and would be cases if had outcome), and b) Hospital controls, within same community as cases (i.e. not another city) but derived from a hospitalized population. Definition of controls: a) If cases are first occurrence of outcome, then it must explicitly state that controls have no history of this outcome. If cases have new (not necessarily first) occurrence of outcome, then controls with previous occurrences of outcome of interest should not be excluded. Comparability of cases and controls on the basis of the design or analysis. Cases or controls are matched in the design and/or confounders are adjusted for in the analysis.

On using Newcastle Ottawa Scale criteria to assess the quality of the three case-control studies, we observed that each of the studies had the cases and controls clearly defined. The comparability was done using proper statistical analysis. But, detailed investigation by including the duration and frequency of smokeless tobacco use with more representative sample size was missed (Table 5).

The subjective quality scoring (Low, Moderate, High) of the included studies was done by two independent reviewers using critical appraisal guidelines proposed by Hermont et al.^26^. The cross-sectional studies were assessed to be of relatively low^36,38^ to moderate quality^37^. With regard to the case control studies, one study was of comparatively low quality37 and the other two were of moderate quality^39,40^, as seen in Table 5. There was no statistical variation between the objective (Newcastle Ottawa Scale) and subjective assessment of the quality of the included studies.

### Meta-Analysis

**Figure 2:**
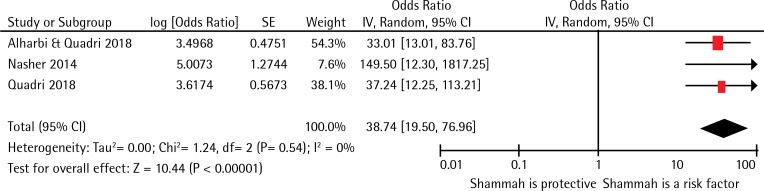
Forest plot demonstrating the relation between oral cancer and SLT in MENA region

Three case-control studies with nearly 534 participants were included in the meta-analysis, and all the studies explored the association between shammah use and oral cancer. Figure 2 demonstrates that the pooled odds ratio for the association between shammah use and oral cancer was 38.74 (95% CI: 19.50–76.96) indicating that the odds for the occurrence of oral cancer among users of shammah were 39 times more than those not using it. The I2 statistic demonstrated that the heterogeneity between the studies was not important (I2=0%, p=0.54). Evaluation of publication bias using Funnel plots or regression-based tests was not possible due to inadequate number of studies included in the meta-analysis.

## DISCUSSION

Cancer of the oral cavity is a matter of great concern due to the global rise in the number of new cases each year^41^. The MENA region is a significant contributor, with oral cancer being the fourth leading cause of death^42,43^. The current systematic review and meta-analysis is the first of its kind in the MENA region to report on the association between smokeless tobacco and oral cancer while assessing the quality of evidence that the published studies provide. The overall finding from this study revealed that there is some association between smokeless tobacco and oral cancer among the residents of the MENA region; and the result is concurrent with an earlier systematic review performed among the population residing in the South Asian region^44^. In two other reviews involving studies from developed nations, the estimated risk of oral cancer among smokeless tobacco users had not been interpreted due to the low number of users^45,46^. These region-specific reviews have been published within the last decade and their findings are of great value. We further support this by stating that the association between the risk factor and disease could be clearly interpreted by controlling for factors such as ethnicity, genetics, lifestyle habits etc. It can also be substantiated by stating that the type of tobacco consumed in one region may not be similar to the one consumed by the inhabitants of another region.

Among the studies included in the current review, shammah was found to be the most commonly reported smokeless tobacco. This powdered tobacco is placed in the buccal or labial vestibule of the oral cavity for a long duration and the extracted juice is swallowed^34^. The included studies also showed that the site of oral cancer lesions was mostly labial mucosa followed by buccal mucosa on the same side of tobacco placement. North Sudan and South Sudan are not included in the MENA region by the World Bank, even though they are part of the North African countries. Some published literatures from these two countries have suggested heavy use of toombak (a form of smokeless tobacco), mostly by the adult population^13,47^.

The studies included in the meta-analyses were heterogeneous and the magnitude of association between shammah and oral cancer was reported through a forest plot. Findings suggested that the odds of shammah users were nearly 39 times higher for oral cancer than those not using shammah. This result is consistent with two other meta-analysis performed elsewhere^48,49^. However, the results from the current study should be interpreted with caution. This is due to the lack of detailed information on the frequency and duration of smokeless tobacco consumed; and also due to the small number of low-to-moderate quality studies included. Another limitation of the current meta-analysis is the lack of publications from other countries in the region; and the included case-control studies have only used hospital-based samples. Thus, findings here are not representative of the general population residing in the MENA region.

Finally, it is to be reiterated that the number of publications arising from the MENA region are fewer in comparison to those from South-East Asia and America^50^. Also, the use of shammah among the people residing in the MENA region, especially females, could be underreported. This can be attributed to existing cultural reasons and that it is illegal to consume smokeless tobacco in countries such as Saudi Arabia^13^. The current study recommends that future researches performed in the region to determine the association should consider the frequency and duration of smokeless tobacco using robust methodology. We also recommend the development of population-based registries in the MENA region that are actively supported by the governmental authorities so that quality data more representative of the population residing in the region are obtained.

## CONCLUSIONS

This meta-analysis indicates an association between shammah and oral cancer in the Middle East and North Africa. Our findings are important for public health practitioners in order to advocate and prevent oral cancer by policing the abuse of shammah in the MENA region.
